# Supplementary material for: Terrain-Perception-Free Quadrupedal Spinning Locomotion on Versatile Terrains: Modeling, Analysis, and Experimental Validation
Source: Front Robot AI. 2021 Oct 26;8:724138. doi: 10.3389/frobt.2021.724138 (PMC8576540; doi:10.3389/frobt.2021.724138)
Supplement: Supplementary file 1 [file DataSheet1.PDF]

Supplementary Material

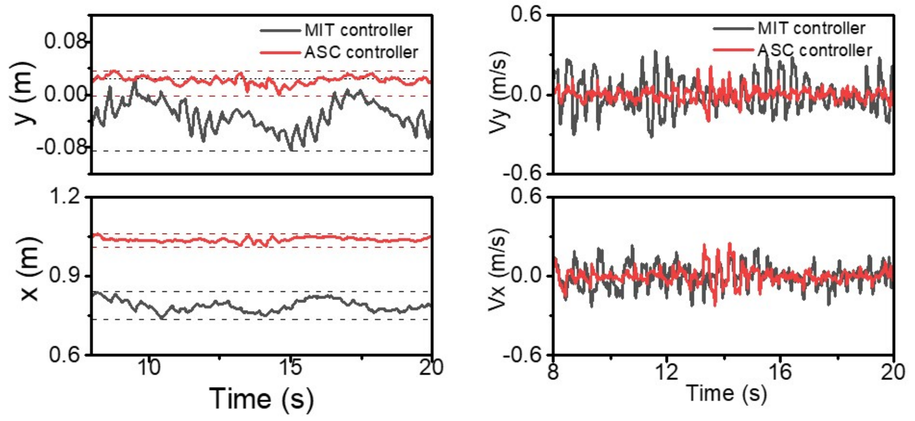

Figure S1. The CoM position drift and velocity in the experiment of spinning on the slope. The black and red lines represent the experimental data of MIT controller and ASC controller respectively.

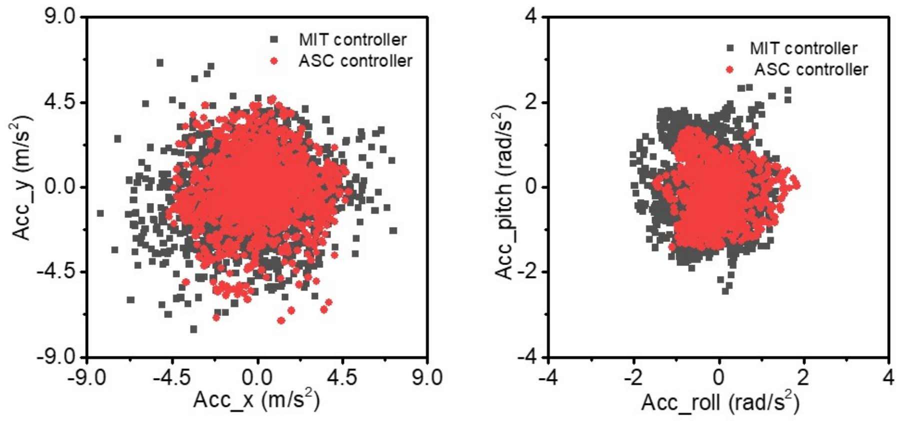

Figure S2. The liner acceleration and the angular acceleration in the experiment of spinning on the slope. The black and red lines represent the experimental data of MIT controller and our ASC controller respectively.

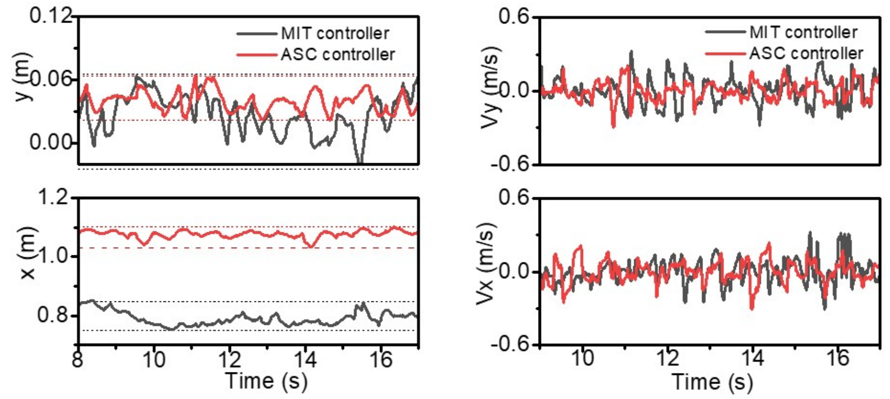

Figure S3. The position drift and velocity in the experiment of spinning on the stairs. The black and red lines represent the experimental data of MIT controller and our ASC controller respectively.

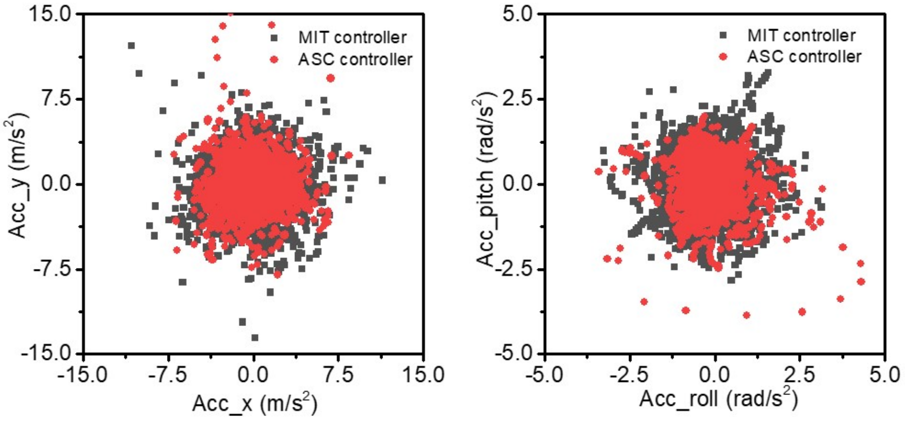

Figure S4. The liner acceleration and the angular acceleration in the experiment of spinning on the stairs. The black and red lines represent the experimental data of MIT controller and our ASC controller respectively.

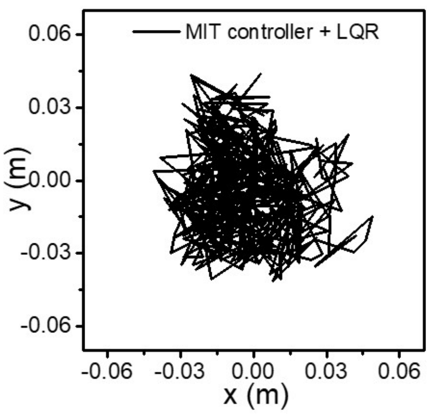

Figure S5. The CoM trajectory of spinning on ground by just using LQR and PSP CoM planner.
